# Robust Finger-vein ROI Localization Based on the 3*σ* Criterion Dynamic Threshold Strategy

**DOI:** 10.3390/s20143997

**Published:** 2020-07-18

**Authors:** Qiong Yao, Dan Song, Xiang Xu

**Affiliations:** Artificial Intelligence and Computer Vision Laboratory, University of Electronic Science and Technology of China, Zhongshan Institute, Zhongshan 528402, China; yaoqiong@zsc.edu.cn (Q.Y.); songdan@zsc.edu.cn (D.S.)

**Keywords:** finger-vein, ROI localization, Kirsch detector, 3*σ* criterion, dynamic threshold

## Abstract

Region of interest (ROI) localization is one of the key preprocessing technologies for a finger-vein identification system, so an effective ROI definition can improve the matching accuracy. However, due to the impact of uneven illumination, equipment noise, as well as the distortion of finger position, etc., these make accurate ROI localization a very difficult task. To address these issues, in this paper, we propose a robust finger-vein ROI localization method, which is based on the 3σ criterion dynamic threshold strategy. The proposed method includes three main steps: First, the Kirsch edge detector is introduced to detect the horizontal-like edges in the acquired finger-vein image. Then, the obtained edge gradient image is divided into four parts: upper-left, upper-right, lower-left, and lower-right. For each part of the image, the three-level dynamic threshold, which is based on the 3σ criterion of the normal distribution, is imposed to obtain more distinct and complete edge information. Finally, through labeling the longest connected component at the same horizontal line, two reliable finger boundaries, which represent the upper and lower boundaries, respectively, are defined, and the ROI is localized in the region between these two boundaries. Extensive experiments are carried out on four different finger-vein image datasets, including three publicly available datasets and one of our newly developed finger-vein datasets with 37,080 finger-vein samples and 1030 individuals. The experimental results indicate that our proposed method has very competitive ROI localization performance, as well as satisfactory matching results on different datasets.

## 1. Introduction

As a promising biometric traits technology, finger-vein identification technology has drawn comprehensive attention in the past decade [1,2,3]. Compared with some other forms of biometrics, such as face, voice, fingerprint, iris, etc., finger-vein shows many superiorities in the aspects of uniqueness, anti-counterfeiting, active liveness, permanence, user-friendliness, and so on. This is due to the fact that finger-vein is hidden underneath the skin’s surface and owns a unique network pattern for the different fingers of different individuals.

Generally, a typical finger-vein identification system consists of four blocks. First, an imaging module is responsible for capturing the finger-vein image. Then, some preprocessing modules are responsible for converting the acquired low-quality images into high-quality ones. After, the feature extraction module is used to obtain discriminant vein feature representation. Finally, the pattern matching module is used to output the identification results. Considering that finger-vein imaging is sensitive to near-infrared (NIR) light with a wavelength range from 700 nm to 1000 nm, the corresponding imaging module usually adopts an NIR light source to penetrate the finger and then outputs a filtered image from a charge-coupled device (CCD) camera [4]. However, due to the low quality of the output finger-vein image, it is hard to use it directly for subsequent feature extraction and matching purposes. Therefore, a series of preprocessing technologies is introduced to obtain more reliable finger-vein images. On the one hand, the finger-vein image acquisition equipment needs to be designed in a very compact and small size, so that it can be applied to related fields, such as security locks, vehicles, etc. In this case, the acquired image often has a low resolution and uneven illumination. Besides, finger veins are underneath the skin layer, leading to the low distinction between the vein network and skin regions in the CCD captured image. Therefore, problems such as noise, blurring, low contrast, uneven gray distribution, etc., are prevalent in the CCD captured finger-vein images, which have had serious impacts on the subsequent feature extraction and pattern matching. To solve these issues, some image processing technologies [5], such as image denoising and image enhancement [6,7,8], are introduced to pursue higher quality image data, so as to gain a better match accuracy for the biometric system itself. On the other hand, the finger-vein pattern itself is dependent on the pressure and amount of blood in the veins, which means the captured finger-vein image will show various patterns (width, brightness, etc.) when the environmental temperature or capturing equipment is changed. To minimize the impact of inner-class differences, the vein pattern should be extracted from the most useful image regions. In this regard, region of interest (ROI) localization is an efficient way to address this issue. In a captured finger-vein image, ROI aims to explore the regions that are only filled with the most salient information, while some useless regions, such as the background, etc., are eliminated. After localizing the ROI of the finger-vein image, feature extraction can be performed on the retained ROI, which not only improves the matching accuracy, but also reduces the computational complexity, since only the smaller and more significant regions are preserved for subsequent processing. As a result, ROI localization plays a critical role in finger-vein identification systems.

After finishing the aforementioned preprocessing tasks, an enhanced and clearer ROI finger-vein image is obtained. Aiming at vein pattern feature representation, a number of handcrafted feature extraction methods from the field of machine learning have been introduced, including structural feature representation (e.g., line structure [4,5,9], curvature structure [10,11], minutiae [12]), statistical feature representation (e.g., local line binary pattern (LLBP) [13,14], local derivative pattern (LDP) [15], moment invariants [16]), and subspace feature representation (e.g., manifold [17], principal component analysis (PCA) [18], two-dimensional principal component analysis (2D-PCA) [19,20], linear discriminant analysis (LDA) [21]). These feature extraction methods can achieve good results on one or more specific finger-vein datasets, which means there are few handcrafted feature extraction methods that are adaptable to all publicly available datasets. One reason comes from the design considerations of these handcrafted feature extraction methods, which are derived from the specific domain knowledge of experts and are difficult to adapt to all situations. Another reason is that the good feature representation depends on an efficient ROI, but how to achieve a robust ROI in all cases is always a challenge. Different from handcrafted feature extraction methods, a deep learning (DL) network can automatically learn high-layer semantic features from large amounts of samples and has also been introduced for finger-vein feature learning. As a kind of representative DL model, convolutional neural networks (CNNs) have been used for finger-vein recognition [22,23,24,25] and verification [26,27] purposes. Corresponding to the handcrafted features, the deep features show robust and stable matching performance irrespective of the quality of the considered finger-vein images. However, most of the CNNs require a threshold ROI image as the input. Hence, the robustness of the ROI localization method will determine the performance of DL-based feature extraction.

Thus, it can be seen that no matter which feature extraction method is adopted, the accuracy relies heavily on the results of image preprocessing, among which a robust ROI localization is particularly important, which is directly related to the stability and accuracy of the matching result. However, uneven illumination, a complicated background, noise, and finger displacement and rotation may contribute to an inaccurate ROI localization. In this regard, this paper proposes a robust finger-vein ROI localization method based on the 3σ criterion dynamic threshold strategy. The main novelties of our work are listed as follows:

- First, the Kirsch edge detector [28] is introduced for edge detection in the acquired finger-vein image. To the best of our knowledge, this is the first time that the Kirsch operator is applied to edge detection of finger-vein images. Compared with some other classical edge detection operators, such as Sobel [24] and Canny [29], the Kirsch operator has a better compromise effect in identifying weak edges and false edges.
- Second, a three-level dynamic threshold, which is based on the 3σ criterion of the normal distribution, is imposed on four parts of the edge gradient image generated by the Kirsch operation, thus obtaining more distinct and complete edge information.
- Third, through labeling the longest connected component at the same horizontal line, two reliable finger boundaries, which represent the upper and lower boundaries, respectively, are defined, and the ROI is localized in the region between these two boundaries.

Our experiments, carried out on both publicly available finger-vein image datasets and our newly developed finger-vein dataset with 37,080 finger-vein samples and 1030 individuals, reveal that the proposed method has very competitive ROI localization performance when compared with other state-of-the-art ROI localization methods, as well as satisfactory matching results on all considered datasets.

The remainder of this paper is organized as follows. Section 2 provides a brief review of related work with ROI localization methods. Section 3 details our proposed finger-vein ROI localization method. Section 4 discusses the experimental results obtained by using different finger-vein datasets. Finally, Section 5 concludes the paper with some remarks and hints at plausible future research lines.

## 2. Related Works

As discussed above, ROI localization is an indispensable preprocessing step for a finger-vein identification system, and much of the related work has been presented in the public literature. These methods focus on the cropped regions of the finger image that have the most salient vein pattern network [30], while some of the less useful regions are removed, thus preserving more efficient vein discrimination information for subsequent feature extraction and matching tasks. Based on the adopted localization strategy, these methods are generally divided into four classes.

The first class of methods is called fixed window-based ROI localization. Basically, the retained ROI rectangle region is much less than the original captured image, and regions that present the background and the fingertip are removed due to few vein vessels [18,31]. In addition, such a fixed window was also performed on a binarized finger-vein image. In [13], the acquired finger-vein image was binarized by using Otsu’s threshold [32] firstly. Then, a fixed window, with a size of 480×160 and centered by using the center pixel of the finger contour region, was chosen as the ROI. In [8], a fixed window, which was defined in terms of the knuckles, was used for ROI localization. In [22], a smaller fixed window with a size of 70×130 was adopted for ROI localization, so as to adapt to the training of neural networks. Compared with the aforementioned methods, it is a rough ROI localization strategy that is hopefully honed by subsequent neural network learning. Admittedly, fixed window-based ROI methods have the advantages of simplicity and intuition. However, this class of methods generally is sensitive to finger displacement, and the obtained ROIs typically have various sizes and need to be normalized to the same size for convenience.

The second class of ROI localization methods mainly combines the threshold strategy and edge extraction technologies to try to outline the accurate edge contour of the finger. In [5], a fixed threshold of 230 was used to generate the binarized image. Subsequently, the Sobel edge image was subtracted from the binarized image, and the final binary mask was obtained after an area thresholding of the resulting images. Finally, the obtained binary mask was used to localize the ROI from the acquired finger-vein image. Similarly, in [29], a global threshold strategy and Canny edge operator were combined to generate a binary mask, and the morphological dilation operator was performed for further edge enhancement. Threshold-based ROI methods are heavily dependent on the threshold selection. If the illumination is even and the gray level difference between the foreground finger area and background is large, the threshold-based ROI methods can work well. However, in practical scenarios, the above conditions are hard to meet.

The third class of methods determines the edge information by using mask calculation. In [33], two horizontal edge detection masks were used to perform convolution on each pixel, and the pixels with the maximum response value were regarded as the finger boundary points. In [34], a comprehensive ROI localization algorithm, including binarization, orientation correction, and regional cutting, was proposed. First, a group of extended masks that were derived from [35] were performed on the upper part and lower part of the captured finger-vein image, and the pixels with the maximum values for each column were regarded as edge points. Then, a shrinking region, which was defined based on the reference line in the second knuckle, was preserved and normalized to be the final ROI image. However, this class of methods usually has expensive computational costs and is sensitive to the broken edges introduced by error binarization.

The fourth class of methods mainly relies on the effective edge detection technology, which is devoted to obtaining more accurate finger edge information. Considering that the finger profile can be approximated as a line of small curvature, the Hough transform was adopted in [36] to detect two peaks that corresponded to the horizontal finger boundary lines, and the ROI was then center cropped with a size of 256×96. For better localizing the phalangeal joint, a sliding window strategy was adopted for the decision of the width and height of the ROI image [30]. A dual-sliding window was adopted in [20] for more accurate localization of the phalangeal joint. Further, in [37], multiple sliding windows including rectangle, disk, diamond, and ellipse were compared, and it was concluded that the ellipse window was more suitable for the detection of the finger joint reference line. In these methods, the coarse finger edges are detected either using edge detection operators (Sobel, Canny, etc.) or using a mask-based edge detection template. In [38], superpixel segmentation (by using simple linear iterative clustering (SLIC) [39]) and the Sobel edge detector were combined to determine accurate finger boundary lines. The finger ROI was localized by the internal tangents of finger boundaries. In [24], the Sobel edge detector and convex hulls were applied to capture the finger region. Then, a 4×20 mask was used to detect the upper and lower boundaries of finger veins. Finally, the ROI was preserved after removing the parts of the fingertip and finger-tail.

In summary, edge detection-based methods represent the main development trend in the finger-vein ROI localization field. However, it is still impossible to obtain accurate edge information directly from the CCD captured finger-vein image by only relying on some edge detection operators (Sobel, Canny, etc.) or mask calculation methods, while it has been proven to be an effective way to combine the binary image obtained by thresholding. With this consideration, we developed a new robust ROI localization method based on the 3σ criterion dynamic threshold strategy. Our proposed method belongs to ROI localization based on the edge detection technology, which is devoted to solving the difficulty of threshold selection in the case of complicated backgrounds. Next, we will elaborate the proposed methods.

## 3. Proposed ROI Localization Method

**Figure 1:**
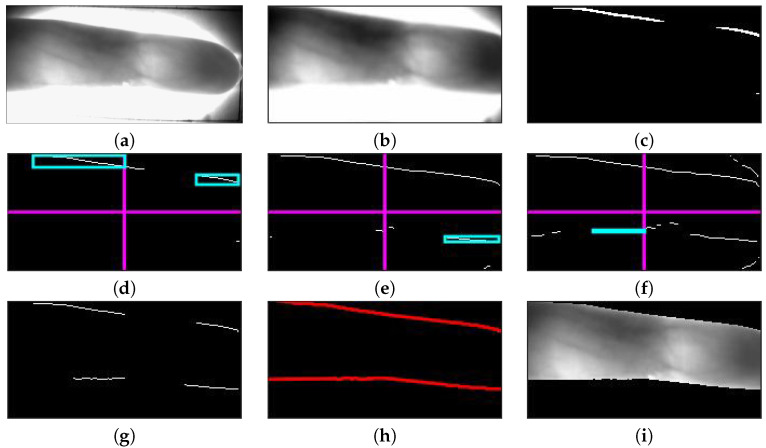
Proposed ROI localization scheme for an example: (**a**) original captured finger-vein image, (**b**) candidate image after filtering and cropping, (**c**) initial binary edge image by threshold of μ+2σ, (**d**) edge of the good quality part by μ+2σ (upper), (**e**) edge of the medium quality part by μ+σ (lower right), (**f**) edge of the poor quality part by μ+0.5σ (lower left), (**g**) final binary edge image, (**h**) binary edge image by interpolation and connection, and (**i**) ROI.

In this section, we describe our proposed ROI localization method, which consists of four main steps. The first step is a filtering and cropping procedure, which is applied on the CCD captured finger-vein image. In the second step, an initial binary image is generated by imposing a threshold of μ+2σ on the Kirsch edge gradient image, in which μ represents the mean gray level of all pixels in the Kirsch edge gradient image and σ represents the standard deviation of the gray level. After these two steps, the initial binary image is divided into four parts: upper-left, upper-right, lower-left, and lower-right. According to the image quality of each part, the three-level dynamic threshold, which is based on the 3σ criterion of the normal distribution, is imposed to obtain more distinct and complete edge information. Here, we divide the quality of the part binary image into three situations: good, medium, and poor (an example is shown in Figure 1. If the quality of the image part is good, this means the edge line is already complete and connected (see Figure 1d), while if the quality of the image part is medium, this means some local weak edges may disappear or the length of the edge line is too short by a certain percentage (see Figure 1e); it will be cut down by a threshold of μ+σ. Lastly, if the quality of the image part is poor (see Figure 1f), the threshold is set to μ+0.5σ. In the last step, two edge lines, which represent the upper and lower boundaries, respectively, are obtained through interpolation and labeling the longest connected component at the same horizontal line (see Figure 1h), and the ROI is localized in the region between these two boundaries (see Figure 1j).

It should be noted that our method is based on some basic assumptions about the captured finger-vein image:

1. The finger is placed horizontal; the finger tip direction is toward the right; if the finger is vertical or the finger tip direction is toward the left, rotate the image in advance.
2. The edges of the finger are two horizontal straight lines; the rotation angle is less than 30 degrees.

**Figure 2:**
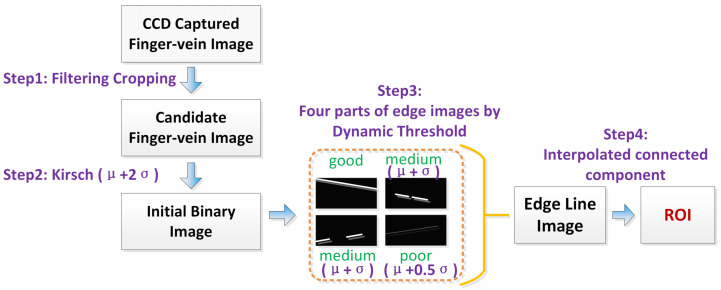
Flowchart of the newly proposed robust ROI localization method.

In the end, an illustrative flowchart of the method is shown in Figure 2. A detailed description of each step is given below.

### 3.1. Preprocessing

As observed from Figure 1a, the CCD captured image contains not only the finger context information, but also much irrelevant information. Therefore, the original image needs to be preprocessed to localize a useful candidate region. In order to eliminate noise, preserve the edge, and smooth the image, we adopted an edge-preserving Gaussian bilateral filter [40] to smooth the original image, and the candidate image was obtained by cropping the marginal areas of the filtered image. Compared with direct edge detection on the original image, more accurate edge information and less noise interferences can be obtained on the filtered candidate image. Finally, both registered and input images were normalized to be the same size for matching purposes.

### 3.2. Kirsch Edge Detection

**Figure 3:**
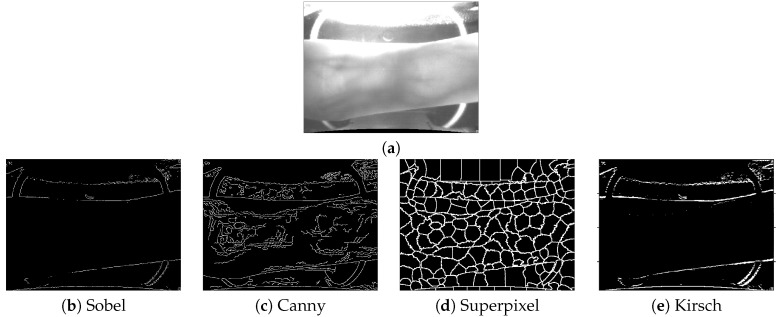
Edge results by using different edge detection operators for an example: (**a**) original finger-vein image, (**b**) Sobel, (**c**) Canny, (**d**) superpixel [38], and (**e**) Kirsch.

The edge detection is a fundamental step in edge-based ROI localization methods, which aims to identify the true finger edges. Among the existing edge detection technologies, Sobel [24], Canny [29], superpixels [38], etc., have been introduced for edge detection of finger-vein images. Generally, the Sobel edge detector can detect edges well with less false edges; however, when the image has plenty of weak edges due to low contrast and uneven illumination, the Sobel edge detector fails relatively more easily; see Figure 3b. Comparatively speaking, the Canny edge detector can recognize more complete edges [29,41], but it also introduces many false edges, which will seriously interfere with ROI localization; see Figure 3c. Superpixels, although having better robustness and device compatibility, still have difficulty obtaining complete and accurate edges when the image quality is poor; see Figure 3d. Thus, it can be seen that a robust edge detect operator, which is insensitive to uneven illumination and noise, is very essential for accurate detection of the boundary edge in finger-vein images. In this regard, we specifically chose the Kirsch detector for edge detection purposes, which shows a better compromise effect in identifying weak edges and false edges; see Figure 3e.

The Kirsch edge operator was proposed by R. Kirsch [28], which adopts eight templates (as shown in Equation (1)) to perform convolutional derivation on each image pixel. These eight templates represent eight directions, and the one with the largest direction response is taken as the edge output.

Compared with other edge detection operators, the Kirsch operator has some obvious advantages. First, it can pursue better edge detection from eight different directions and has a better effect in eliminating noise and preserving details. In addition, threshold selection is relatively more convenient in the Kirsch edge gradient image, and a clearer binary image can be obtained. However, the Kirsch algorithm itself also has certain deficiencies: it has a heavier computational burden, and if the quality of the captured image is poor (due to uneven illumination and noises), the edges obtained by using the Kirsch operator have less continuity; some weak edges will be missed. In this case, the determination of the optimal threshold is different from the image; even every part of the image needs to adopt different thresholds to extract edge information. To solve these issues, we propose to combine the dynamic threshold strategy with the Kirsch operator, so as to obtain more accurate edge information.

(1) $$\begin{array}{c} \left[\begin{array}{ccc} 5 & 5 & 5\\ -3 & 0 & -3\\ -3 & -3 & -3 \end{array}\right]\left[\begin{array}{ccc} -3 & 5 & 5\\ -3 & 0 & 5\\ -3 & -3 & -3 \end{array}\right]\left[\begin{array}{ccc} -3 & -3 & 5\\ -3 & 0 & 5\\ -3 & -3 & 5 \end{array}\right]\left[\begin{array}{ccc} -3 & -3 & -3\\ -3 & 0 & 5\\ -3 & 5 & 5 \end{array}\right]\\ \begin{array}{cccccccccccccc} \; & \; & M1 & \; & \; & M2 & \; & \; & M3 & \; & \; & \; & M4 & \; \end{array}\\ \left[\begin{array}{ccc} -3 & -3 & -3\\ -3 & 0 & -3\\ 5 & 5 & 5 \end{array}\right]\left[\begin{array}{ccc} -3 & -3 & -3\\ 5 & 0 & -3\\ 5 & 5 & -3 \end{array}\right]\left[\begin{array}{ccc} 5 & -3 & -3\\ 5 & 0 & -3\\ 5 & -3 & -3 \end{array}\right]\left[\begin{array}{ccc} 5 & 5 & -3\\ 5 & 0 & -3\\ -3 & -3 & -3 \end{array}\right]\\ \begin{array}{cccccccccccccc} \; & \; & M5 & \; & \; & M6 & \; & \; & M7 & \; & \; & \; & M8 & \; \end{array} \end{array}$$

We aim at the edge detection of finger-vein images, and if the hypothesis holds that the fingers are placed horizontally, we only need to detect two horizontal direction edges. Therefore, only two templates of Kirsch’s horizontal directions (M1 and M5) are required for horizontal-like edge detection. These two Kirsch horizontal direction templates can not only eliminate the interference of non-horizontal boundary lines, but also improve the calculation speed. Concretely, the Kirsch horizontal gradient magnitude can be calculated by using Equation (2).

(2) $$G_{Kirsch}=\max\left\{M_{k}*A\right\},k=1,5$$

### 3.3. Boundary Line Detect

Edge line detection is a classical research issue in computer vision [42]. A common approach is to do edge detection (e.g., Canny edge detector [43]) first and then perform a Hough transform [44], thus hoping to extract all boundary lines that contain a number of edge points exceeding a threshold. It is based on the assumption that every edge point in the edge map is transformed to all possible lines that could pass through that point. However, the Hough transform easily generates false lines in the textured regions with high edge density (see the slanted lines in Figure 1d). In addition, the Hough transform has some inadequacies, such as repeated detection of the spatial peak parameters, complex calculation, slow processing speed, etc. Especially for a skewed finger, where the boundary edge points form a continuous curve, it is difficult to detect the boundary with the Hough transform.

Besides the Hough transform, the strategy of labeling the longest connected component in a binary edge image is another alternative to localize the finger boundary well. When the quality of the image is good, the boundary edge points are continuous, and it is simple and fast [45,46]. However, in practical scenarios, due to uneven illumination, the binary edge points are discontinuous, leading to the deficiency of the connected component.

After comprehensive comparison, in our work, we chose the connected component to detect the boundary edge line, because it is closer to real-time and robust. As described in Section 3.2, for the Kirsch detect operator, a higher threshold is more capable of detecting the stronger and clearer edges, while a lower threshold is more capable of detecting the more complete weak edges. However, the determination of the optimal threshold relies on the actual quality of the image, and even different image parts will need different thresholds in one poor-quality image.

The normal distribution, also known as the Gaussian distribution, has two parameters, the mean μ and variance $\sigma^{2}$. For random variables that are subject to the normal distribution, the mean value determines the overall position of the normal distribution. The closer the variable is to the mean value, the greater the probability of the variable, and the variance of random variables determines the amplitude of the normal distribution. Therefore, the normal distribution is often described as $N(\mu ,\sigma^{2})$. In addition, for random variables subject to the normal distribution, the smaller the variance is, the more concentrated the distribution of the random variables.

**Figure 4:**
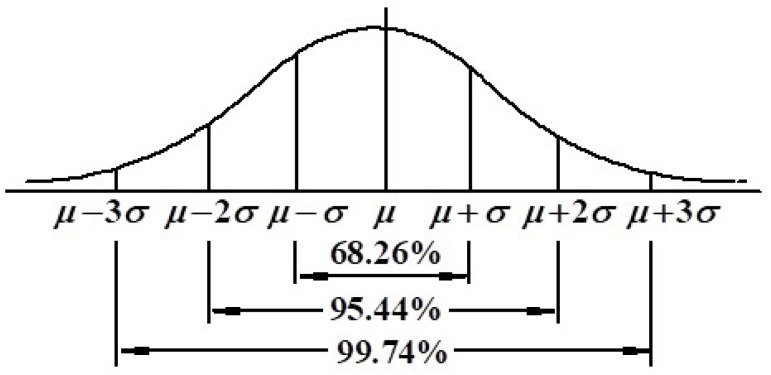
The 3σ criterion of the normal distribution.

The 3σ criterion is suitable for processing samples with normal and nearly normal distributions and requires a large amount of data. As shown in Figure 4 for the 3σ criterion, the probability of the numerical distribution in (μ−σ, μ+σ) is 0.6827; the probability of the numerical distribution in (μ−2σ, μ+2σ) is 0.9544; and the probability of the numerical distribution in (μ−3σ, μ+3σ) is 0.9974. Because the data distributed in (μ−3σ, μ+3σ) accounts for 99.74% of the total data, most of the data distribution characteristics conform to the normal distribution 3σ criterion.

For a finger image with a size of m×n, the boundary edge only employs two rows representing the upper and lower edges where the gradient changes the most significantly. Compared to the whole image pixels, the number of boundary pixels is few. In an ideal situation, when edge detection is carried out, there will be no other edges in the binary edge image except the boundary edges. With this consideration, it is reasonable to regard the gradient distribution in the Kirsch gradient image as a normal-like distribution.

According to the 3σ criterion, the initial threshold is set to μ+2σ, then only 2.28% points are reserved to be the candidate boundary edge. If the image quality is good, the threshold is appropriate for strong edges. For an image with poor quality, the illuminations of every part of image are uneven, and some weak edges cannot be detected; in this case, the threshold can be cut off at μ+σ, then the number of candidate points is enlarged to 15.87% of the whole image. If part of the image edge detection still does not satisfy the requirement, the threshold can be further cut off at μ+0.5σ; thus, the number of candidate points is enlarged to 30.85%. as shown in Figure 4. The whole detection process is carried out by automatically judging the length of the connected components of each part image, if the length of connected edge lines is greater than a preset value *L*, a smaller threshold is not required, and this part image is denoted as good quality. On the contrary, if the length of the connected edge lines is less than the preset value *L*, smaller thresholds are needed for further processing. Here, the value of *L* can be set to the range of [1/3,1/2] of the length of the part image; at this time, there is enough edge information to generate the complete boundary lines.

### 3.4. ROI

The image is divided into four parts: upper-left, upper-right, lower-left, and lower-right. In each part of the image, the horizontal Kirsch operator is performed on it by the higher threshold μ+2σ; thus, four binary edge sub-images can be obtained respectively. In this step, in order to label the connected component on the same horizontal position, we chose the longest connected component as it is more likely to be the finger boundary in this part. Concretely, if the image quality is good, which means the illumination is even, the binary image by threshold μ+2σ is acceptable to find the complete boundary edge. While there always exists uneven illumination and noises, some local weak edges cannot be detected. The threshold is cut down to μ+σ on the local part to find the weak edges. If the length of the local weak edge is too short (here, the threshold was set to half the width of the part), the threshold is cut down to μ+0.5σ again on the local part. Finally, the binary edge image is obtained by interpolating and connecting the line segments to form the final boundary edge, and the ROI is localized in the region between the upper and lower boundary.

For illustration purposes, Figure 1 also presents the results of edge line detection and ROI localization by using a poor-quality image under uneven illumination. First, the candidate image, after filtering and cropping of the original captured finger-vein image (Figure 1a), is presented in Figure 1b, Second, the μ+2σ threshold is performed on a Kirsch gradient image (which is derived from two horizontal Kirsch templates), so as to obtain an initial binary edge image, as shown in Figure 1c. After, the binary edge image is divided to four parts. For the upper-left and upper-right parts (Figure 1d), there is enough long connected component that is longer than the half width of the corresponding part, so the edge lines in these two parts can be directly labeled. However, as observed from Figure 1d, there is nothing that can be labeled in the lower-left and lower-right parts. Therefore, we gradually cut down the threshold to μ+σ (Figure 1e); at this moment, enough long connected component can be labeled in the lower-right part, while still nothing can be labeled in the lower-left part. Therefore, we again cut down the threshold to μ+0.5σ, and the connected component can emerge in the lower-left part eventually (Figure 1f). We integrate the detection results of the four parts and obtain the edge line image as shown in Figure 1g, then the complete boundary lines by interpolation and connection are shown in Figure 1h. Finally, the ROI is localized in the region between the upper and lower boundary edge line, as shown in Figure 1i.

## 4. Experimental Analysis

To ascertain the effectiveness of our proposed ROI localization method, we carried out four experiments on the four different finger-vein datasets, which were acquired from different sensors. The first experiment aimed to assess the overall quality of image samples from different datasets. The second experiment presented the robustness of our ROI localization method, in which three typical image samples representing different quality levels (good, medium, poor) were selected from different datasets. The third experiment carried out a comparative analysis between our ROI localization method with two commonly used methods, including the threshold-based method [5] and the mask-based method [33]. The fourth experiment conducted a comparative assessment of the matching results. Finally, we would like to emphasize that all the experiments were conducted by using a desktop PC equipped with an Intel Core i7 CPU (at 3.6 GHz) and 32 GB of RAM.

### 4.1. Datasets

Table 1 shows the related properties of the four finger-vein datasets used in our experiments. Among them, the first three datasets are publicly available, namely HKPU [5], MMCBNU_6000 [34], and FV-USM [47], respectively. Moreover, in order to assess the effectiveness on a larger dataset, we developed a new finger-vein dataset (namely “ZSC-FV”) by using the acquisition equipment of Beijing Yannan Tech Co., Ltd, which contains 37,080 finger-vein image samples. The details of these datasets are described as follows:

In the HKPU [5] dataset, finger-vein images from 105 individuals were acquired in two separate sessions with an average of 66.8 days. In each session, every individual provided 6 image samples from two fingers, while the other 51 individuals had one single session of acquired data. There was an obvious rectangle frame in the captured image, and the existence of shadows would interfere with correct judgment. In the following experiments, thirty pixels from the upper border, 10 pixels from the lower border, 30 pixels from the left border, and 50 pixels from the right border were removed, then all the cropped images were scaled to half of the original size by the bicubic method. Finally, the size of HKPU images was fixed to 109×217.

The MMCBNU_6000 [34] dataset contains finger-vein images from 100 individuals from only one session. Each individual provided 6 fingers, which was repeated 10 times to obtain 10 finger-vein images. The overall quality of this dataset is good, and the background is almost pure black. In the following experiments, five pixels were removed from borders, and the candidate region was scaled to one quarter of the original. Finally, the size of MMCBNU_6000 images was fixed to 118×158.

In the FV-USM [47] dataset, finger-vein images from 51 individuals in two sessions were captured. Each individual provided 10 images of 4 fingers. The orientation of the fingertip was downward, and there were too many black areas without any information. In the following experiments, the image was rotated $90^{\circ }$ anti-clockwise, followed by 150 pixels from the upper and lower borders, 5 pixels from the left border, and 70 pixels from the right border being removed. Finally, the candidate region was scaled to half of the original, thus obtaining a size of 171×203.

In our self-built ZSC-FV finger-vein dataset, a total of 37,080 finger-vein images were collected from 1030 undergraduate volunteers. The ages of these individuals covered a range from 18 to 22 years old. Each provided 6 image samples from the index, middle, and ring fingers of both hands. The acquisition process was completed in an indoor environment with different illumination conditions. All images were in 8 bit BMP format, 256 grayscale, and had a size of 384×512. The capturing device was manufactured by Beijing Yannan Tech Co., Ltd. The fingertip was oriented to the right and outside the image region. The captured background was very complicated, and the noise regions had a high edge density. In the following experiments, twenty pixels from the borders were removed, and the candidate region was scaled to half of the original size by the bicubic method. Finally, the size of ZSC-FV images was 173×237.

**Figure 5:**
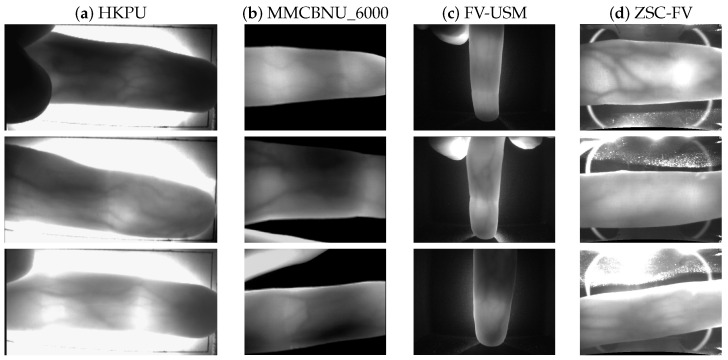
Three different qualities of images from the four different datasets. The first row corresponds to the good quality, the second row to the medium quality, and the third row to the poor quality. (**a**) HKPU, (**b**) MMCBNU_6000, (**c**) FV-USM, and (**d**) ZSC-FV.

Here, we specially picked out three image samples from each dataset, representing the qualities of good, medium, and poor, respectively (as shown in Figure 5). It can be observed from Figure 5 that the image size, quality, and background were all different in the four datasets, and some preprocessing such as cropping and resizing was carried out before using them. In Figure 5, the first row is the image samples of good quality, and we could obtain the complete boundary edges by using only one threshold of μ+2σ. The second row is the image samples of medium quality, which were affected to some extent by the uneven illumination. Thus, in this case, we needed to resort to the second threshold of μ+σ to obtain the parts of the boundary edges. Finally, the third row shows the poor-quality image samples, in which the weak edges in some parts of image needed to be obtained by the third threshold of μ+0.5σ.

### 4.2. Analysis of the Overall Quality of the Four Datasets

Under the condition of even illumination and less noise, the acquired finger-vein images were generally of better quality, and the binary edge was easy to detect. However, when the illumination became uneven and the noise increased, the corresponding binary edge became hard to detect. After using the two horizontal templates of Kirsch to perform convolution on the image, although a higher threshold could obtain more real edges, the completeness was difficult to guarantee, while for the lower threshold, even though it could yield more edges, these edges were also adulterated with many false edges.

For an image with a size of m×n, we could assume that all pixels’ gradients conformed to an approximate normal distribution, where the pixels on the required boundary edges had the most significant gradients. Therefore, relative to the whole image, it could be considered that the pixels on these two edge lines were approximately on the right side of μ+2σ, as seen from Figure 4. According to the 3σ criterion of the normal distribution, the binary edges of the high-quality images can be completely obtained by threshold μ+2σ, while for the medium-quality image, the thresholds of different parts were different, and the lower threshold μ+σ was needed to get the local binary edges. Furthermore, for some poor-quality images, the binary edge of some parts of the image could be obtained by an even lower threshold of μ+0.5σ.

We applied the proposed method to the above four datasets, and the corresponding statistical results of the overall quality are shown in Table 2. For the HKPU dataset, twenty-nine-point-three-one percent of the images could achieve the binary edges by only one threshold μ+2σ, and these part of the images were claimed as good quality. Forty-eight-point-five-three percent of the images needed a lower threshold μ+σ in some local parts after the first threshold μ+2σ, and these part of the images were claimed as medium quality. Finally, twenty-two-point-sixteen percent of the images needed an even lower threshold of μ+0.5σ to obtain a locally complete binary edge after performing two thresholds of μ+2σ and μ+σ in turn, and these part of images were claimed as poor quality. Therefore, the overall quality of the HKPU dataset was poor due to uneven illumination, noise, shadow, covered, rotation, and displacement. The quality of both the MMCBNU_6000 and ZSC-FV datasets was good; most of fingers were horizontal, with even illumination; over 94% of the images could achieve complete binary edges by one threshold. The difference between these two datasets was that the background of MMCBNU_6000 was almost pure black, while the background of ZSC-FV was more complicated; some high density noise areas were easily mistaken as edges. The quality of FV-USM was between HKPU and MMCBNU_6000. The illumination on the boundary was uneven, leading to the boundary lines between the finger and the background being less clear.

The statistical results in Table 2 indicate that, due to the factors of the collection equipment and environment, image samples of different quality commonly existed in each dataset. Thus, only one single threshold had difficulty to meeting the processing of all image samples. In this regard, the threshold strategy we put forward fully considered this reality and adopted a three-level dynamic threshold, so as to better solve the problem of boundary line detection of different qualities of finger-vein images. In addition, we report the average computation time of various datasets in Table 3. Here, the computation time was proportional to the size and quality of the dataset. The average time of the Kirsch operator was between 27.4 ms and 64 ms. The average time of labeling the longest connected component on a good-quality image was between 0.9 ms and 11 ms. Some medium-quality images needed a lower threshold to find the local connected component, and such an additional detection overhead resulted in the average computation time increasing to between 50.8 ms and 64.8 ms. Some poor-quality images even needed a third lower threshold to find the local connected component, and the results in the average computation time expanded to between 92.7 ms and 129 ms.

### 4.3. Typical ROI Extraction Results by the Proposed Method

To assess the robustness of the proposed ROI localization method, we specially chose three typical image samples representing different qualities from each dataset and present the final ROI results, as well as the relevant intermediate results. The chosen samples had obvious distinctions in the aspects of image size, gray level, and the noise of the background. Moreover, finger displacement also existed in some images.

**Figure 6:**
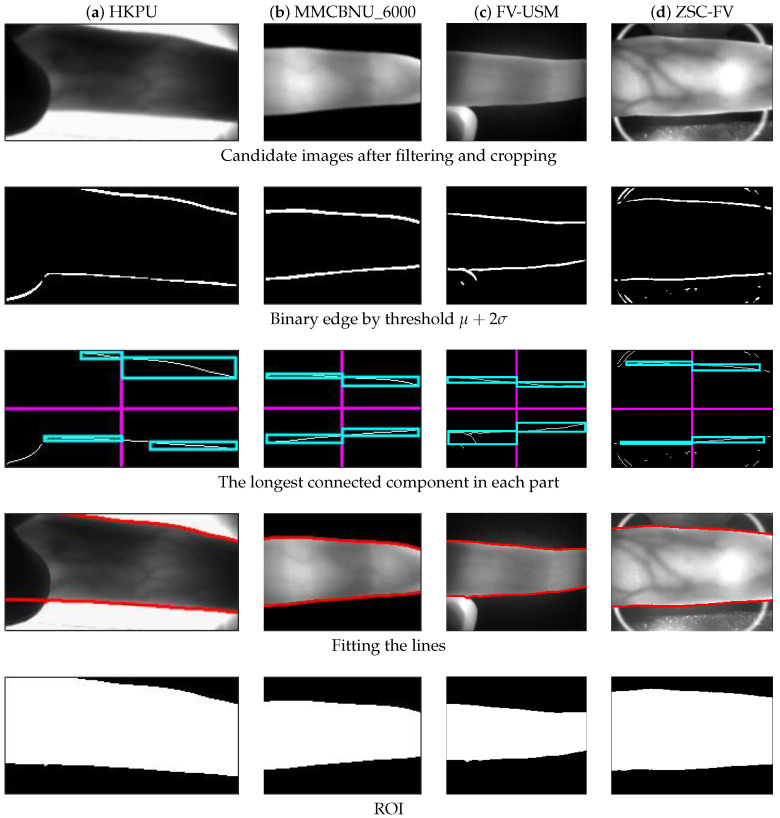
Processing results of some good-quality images from the four datasets, respectively. (**a**) HKPU, (**b**) MMCBNU_6000, (**c**) FV-USM, and (**d**) ZSC-FV.

Figure 6 shows the ROI results of four good quality samples from the four different datasets, one for each column. The first row shows the candidate image after filtering and cropping (as described in Section 3.1). The second row shows the binary Kirsch edge image by threshold μ+2σ. The third row shows the found longest connected component in each part of the image. In this case, since all image samples had good quality, the boundary edges were almost complete. Finally, the fourth row exhibits the fitting result, and the fifth row exhibits the ROI mask between the upper and lower lines. It can be observed from the results of the last two rows that the two extracted boundary lines matched the finger contour very well, thus separating the finger region from the background. Therefore, we could conclude that the obtained ROI results were visually accurate.

**Figure 7:**
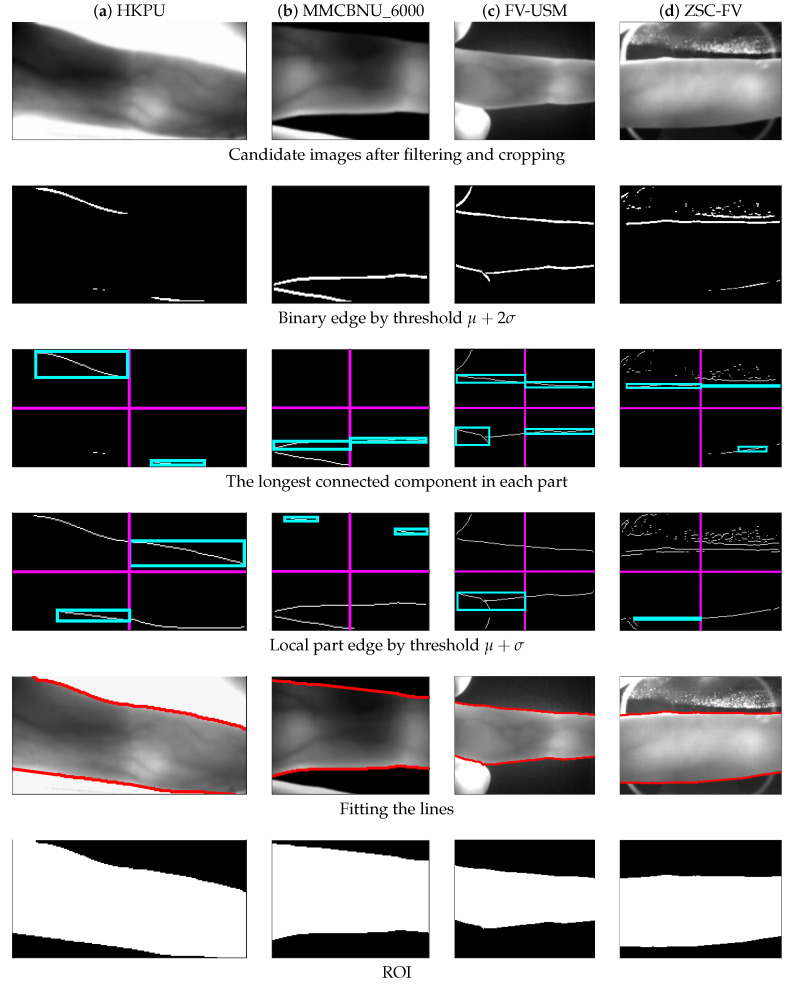
Processing results of some medium-quality images from the four datasets, respectively. (**a**) HKPU, (**b**) MMCBNU_6000, (**c**) FV-USM, and (**d**) ZSC-FV.

Figure 7 shows the ROI results of four medium-quality samples from four different datasets, one for each column. As can be observed from the second row of Figure 7, due to uneven illumination and noises, some parts of the image could not obtain a complete binary edge by one threshold μ+2σ, except for the FV-USM dataset. Therefore, we cut down the threshold to μ+σ for further boundary edge detection, and the corresponding results are shown in the fourth row of Figure 7. Compared with the results of the second row, some of the missing edges in parts of the image were found. Finally, the results of fitting lines from the fifth row and the ROI mask results from the sixth row both proved the effectiveness of the proposed method.

**Figure 8:**
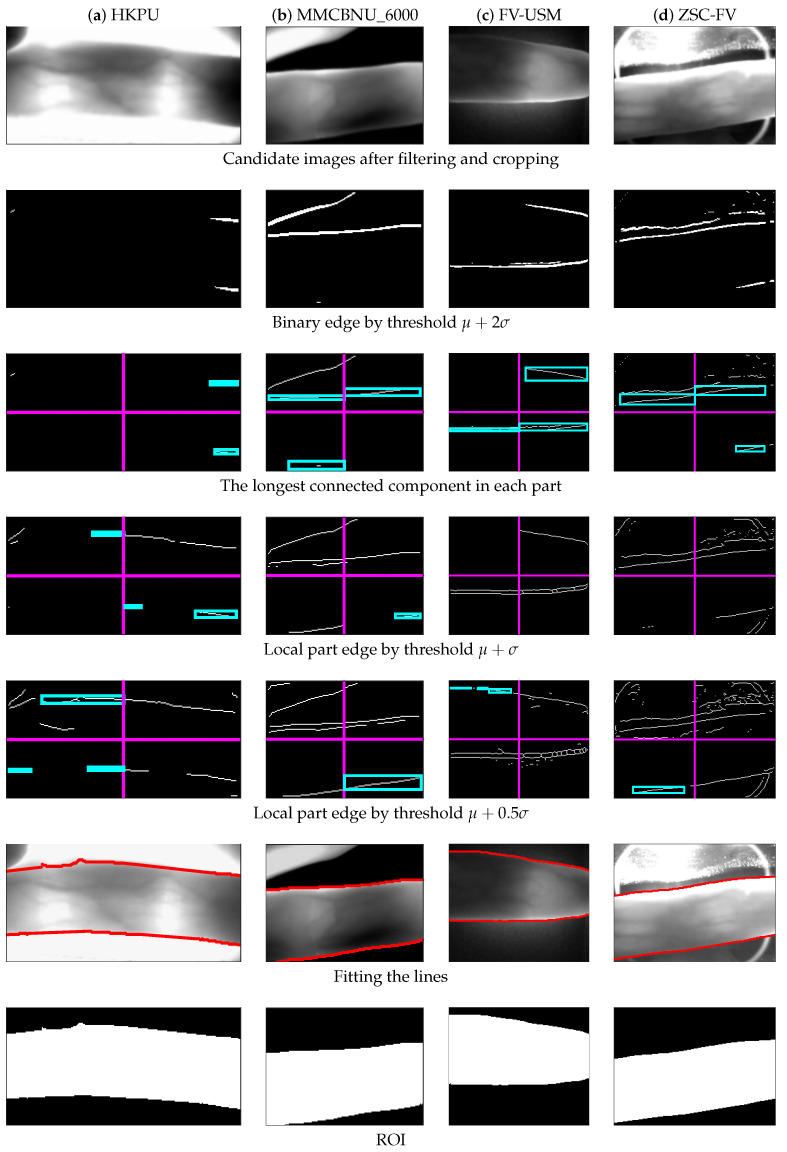
Processing results of some poor-quality images from the four datasets, respectively. (**a**) HKPU, (**b**) MMCBNU_6000, (**c**) FV-USM, and (**d**) ZSC-FV.

Figure 8 shows the ROI results of four poor-quality samples from four different datasets, one for each column. As can be observed from the second row, the obtained binary edges were very incomplete, especially for HKPU. After performing the second threshold of μ+σ, some parts of the image still could not get a complete binary edge (as shown in the third and fourth rows of Figure 8). Again, we cut down the threshold to μ+0.5σ in these local parts, so as to get the missing edges, and the corresponding results of local edges are shown in the fifth row of Figure 8, while the results of the fitting lines and the ROI mask are shown in the sixth and seventh rows, respectively.

After comprehensively considering the ROI localization results in the above three circumstances of Figure 6, Figure 7 and Figure 8, we could draw the conclusion that the proposed 3σ criterion dynamic threshold strategy could effectively extract accurate finger-vein ROIs, no matter the quality of the images that were derived from different sensors.

### 4.4. Comparison of Different ROI Methods

In this experiment, we compared our ROI method with the threshold-based method [5] and the mask-based method [33]. For the threshold-based method, the threshold for HKPU was set to 230, following the recommendation in [5], while the thresholds for the other three datasets were set by Otsu’s method [32]. For the mask-based method, the ROIs were extracted directly by running the program at https://ww2.mathworks.cn/matlabcentral/fileexchange/35752-finger-region-localisation.

**Figure 9:**
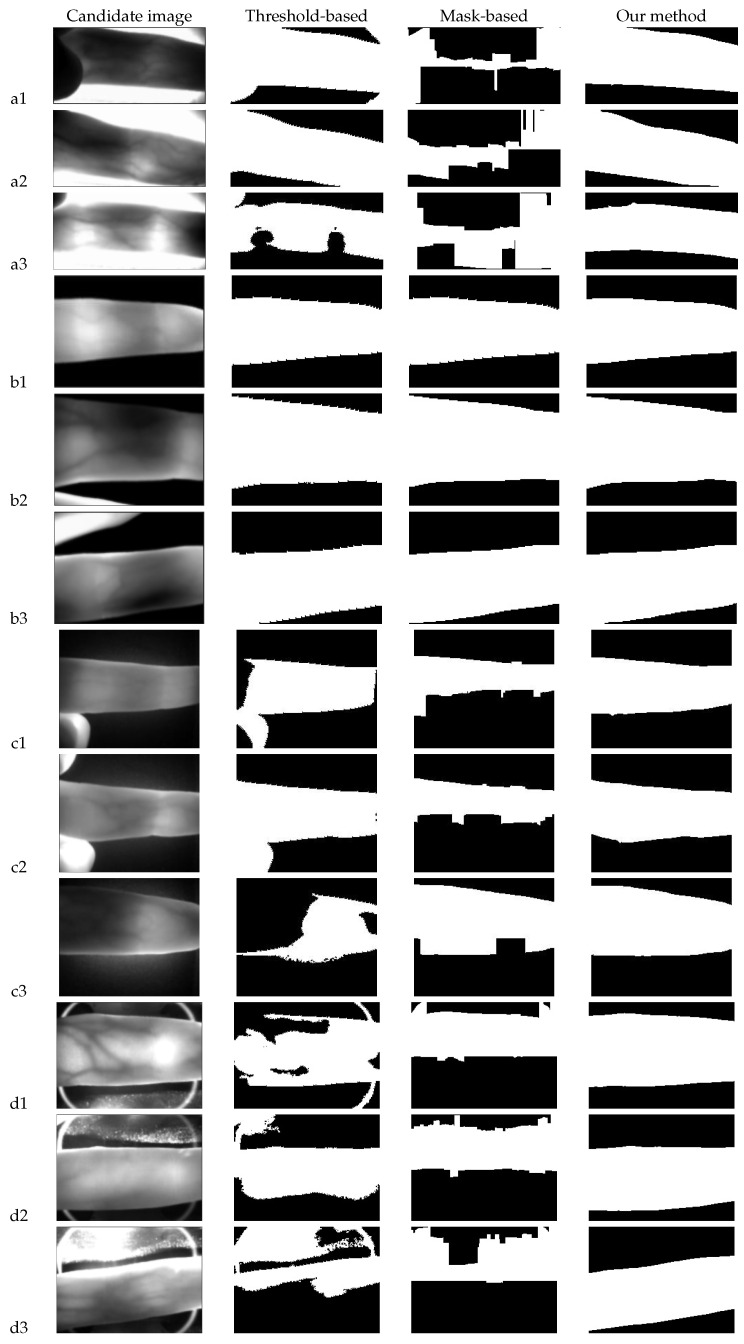
Comparison of the results of our proposed method with the threshold-based method and the mask-based method. (**a1**–**a3**) are the results from the HKPU dataset with good-, medium-, and poor-quality images. (**b1**–**b3**) are the results from the MMCBNU_6000 dataset with good-, medium-, and poor-quality images. (**c1**–**c3**) are the results from the FV-USM dataset with good-, medium-, and poor-quality images. (**d1**–**d3**) are the results from the ZSC-FV dataset with good-, medium-, and poor-quality images.

Figure 9 exhibits the ROI results of the comparison methods on the 12 finger-vein images, and these images are shown in Figure 5. The first column represents the candidate image, and for the sake of fairness, all three comparison methods were applied to this same image. The second column is the ROI results of the threshold-based method; the third column is the ROI results of the mask-based method; and the fourth column shows the ROI results of our proposed method.

As can be observed from the second column of Figure 9, the threshold-based method could work well on both the HKPU and MMCBNU_6000 datasets, since the captured images were relatively clean and the difference in brightness between the finger region and background was relatively distinct. For the FV-USM and ZSC-FV datasets, the threshold-based method was shown to not be suitable. This was due to the fact that in the FV-USM and ZSC-FV datasets, the background regions exposed a large number of unexpected brightness variations, and this phenomenon severely occurred near the boundary of the finger region. The third column of Figure 9 exhibits the ROI results by using the mask-based method. These ROI results were obtained by choosing the first two pixels with the maximum response value of each column in the edge gradient image to locate the finger boundary lines. From the results of the third column, we could conclude that the mask-based method worked well on the MMCBNU_6000 dataset, but not so well on the other three datasets. This was due to the fact that the mask-based method was very sensitive to noise; for example, the noise boundary was likely to be wrongly regarded as a finger boundary, since the noisy pixels may have the maximum response value. The fourth column of Figure 9 exhibits the results of the ROI mask by our proposed method. The experimental results indicated that the proposed method was robust to different qualities of images from different capturing devices.

### 4.5. Analysis of Matching Performance

**Figure 10:**
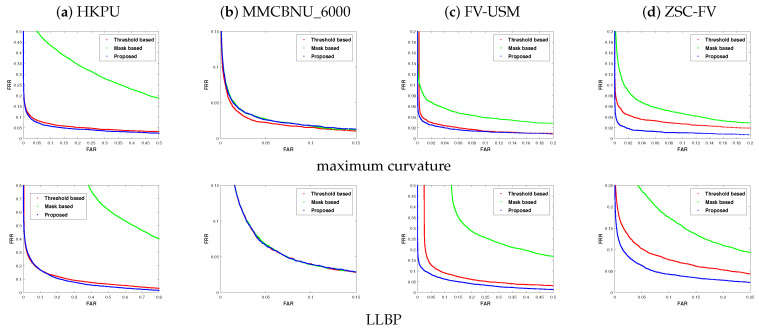
Comparison of the ROC curves of different kinds of ROIs, obtained by using maximum curvature [10] and LLBP [13] features, respectively. (**a**) HKPU, (**b**) MMCBNU_6000, (**c**) FV-USM, and (**d**) ZSC-FV.

In this experiment, we analyzed the matching performance of the proposed method with the other ROI localization methods. Here, two representative ways of feature extraction, including maximum curvature [10] and LLBP [13], were adopted for fairness, which means the different ROI results from the threshold-based method [5], the mask-based method [33], and the our proposed method were then input for the same feature extraction. The ROC curves for the corresponding datasets are illustrated in Figure 10, and the corresponding quantitative matching results are shown in Table 4. It should be noted that the matching performance relied heavily on multiple aspects, such as the image enhancement method, the parameters of the feature extraction method, the distance metric, the classifier, etc. However, the focus of this paper is to exploit a robust finger-vein ROI localization method, other than an effective feature extraction method or an effective distance metric method.

The matching score between a registered and an input image was measured based on the robust template matching strategy proposed in [4]. The false rejection error rate (FRR) refers to the error rate of falsely rejecting the authentic matching attempt (recognizing an image of the same class as that of the enrolled finger-vein) as the imposter class. Conversely, the false acceptance error rate (FAR) refers to the error rate of falsely accepting (as the enrolled finger-vein) an imposter matching attempt (recognizing an image of a class different from that of the enrolled finger vein) as a genuine class. In general, FRR and FAR were in a trade-off relationship depending on the threshold for the finger-vein recognition; the EERwas the case where FRR equaled FAR.

If the dataset included *k* number of images for each of *m* fingers from *n* individuals, the total number of images was n×m×k. As all the fingers were reported to have different vein patterns, the number of classes was n×m. Consequently, because *k* number of images existed per class, the number of genuine matches per class was $C_{k}^{2}$ and the number of total matches $C_{n\times m\times k}^{2}$. Among, the number of genuine tests was $n\times m\times C_{k}^{2}$, and the number of imposter tests was $C_{n\times m\times k}^{2}-n\times m\times C_{k}^{2}$.

For the HKPU dataset, one-hundred and five individuals were acquired in two separate sessions, while another 51 individuals had only one single session of acquired data. Therefore, in the matching experiment, the first session was selected that contained six images for each of two fingers from 156 individuals. The total number of images was 1872 (2×156×6), the number of genuine tests $312\times C_{6}^{2}$, and the number of imposter tests $C_{1872}^{2}-312\times C_{6}^{2}$. As for the three other datasets, three-hundred and twelve fingers were captured; six images per finger were randomly selected, and 10 repeated experiments were conducted.

It can be observed from Table 4 and Figure 10, under the same feature extraction method and for the same dataset, that our proposed ROI localization method obtained better results than the threshold-based and mask-based ROI methods, which again proved the robustness of the proposed method.

## 5. Conclusions

In this paper, we present a robust finger-vein ROI localization method, which is based on the 3σ criterion dynamic threshold strategy. In our method, the Kirsch edge detector is first recommended in edge-based ROI detection, and then, a new dynamic threshold strategy according to the 3σ criterion of normal distribution is proposed for more accurate identification of boundary lines. After dividing the image into four parts, the threshold is set iteratively according to the quality of each local part. Our experimental results indicate that the newly developed robust ROI localization method is very adaptable to different datasets. As with any new method, there are some unresolved issues that may present challenges over time. Specifically, our method requires a three-level dynamic threshold strategy, so as to obtain accurate and complete boundary edges. In this case, the computational complexity increases invisibly. Considering that the threshold setting is always a hot issue in the finger-vein identification community, it is acceptable to sacrifice a certain computational speed for more accurate localization results. In the future, we will develop optimized versions to enhance the practical application of our proposed method.

## Figures and Tables

**Table 1:** Finger-vein datasets.

Dataset	Number of Individuals	Finger	Hand	Images Per Finger	Session	Finger Class	Total Images	Image Size
HKPU [5]	156	middle	left	6/12	2	312	3132	513×256
MMCBNU_6000 [34]	100	middle, ring	left, right	10	1	600	6000	480×640
FV-USM [47]	123	middle	left, right	12	2	492	5904	640×480
ZSC-FV	1030	index, middle, ring	left, right	6	1	6180	37,080	384×512

**Table 2:** Statistics of the number of image samples with different qualities for the four datasets.

Dataset	Good	Medium	Poor
HKPU	918 (29.31%)	1520 (48.53%)	694 (22.16%)
MMCBNU_6000	5687 (94.78%)	26 (4.33%)	53 (0.88%)
FV-USM	4926 (83.43%)	802 (13.58%)	176 (2.98%)
ZSC-FV	35,090 (94.63%)	1778 (4.8%)	212 (0.57%)

**Table 3:** Statistics on the average computation time (milliseconds) for the four datasets.

Dataset	Kirsch Detection	Good	Medium	Poor	Image Size
HKPU	34.4	8.4	64.8	129.0	109×217
MMCBNU_6000	27.4	8.5	52.0	104.4	118×158
FV-USM	51.3	0.9	50.8	92.7	171×203
ZSC-FV	64.0	11.0	59.0	108.6	173×237

**Table 4:** EERscalculated on different kinds of ROIs by using maximum curvature [10] and local line binary pattern (LLBP) [13] features, respectively.

Feature	ROI	HKPU	MMCBNU_6000	FV-USM	ZSC-FV
maximum	Threshold-base	0.0752	0.0292	0.0276	0.0367
curvature	Mask-based	0.2871	0.0335	0.0512	0.0585
	Our method	0.0668	0.0338	0.0243	0.0190
	Threshold-base	0.1420	0.0590	0.0940	0.0843
LLBP	Mask-based	0.5631	0.0596	0.2539	0.1412
	Our method	0.1403	0.0593	0.0727	0.0570
